# Spices in the Apiaceae Family Represent the Healthiest Fatty Acid Profile: A Systematic Comparison of 34 Widely Used Spices and Herbs

**DOI:** 10.3390/foods10040854

**Published:** 2021-04-14

**Authors:** Ramesh Kumar Saini, Awraris Derbie Assefa, Young-Soo Keum

**Affiliations:** 1 Department of Crop Science, Konkuk University, Seoul 05029, Korea; saini1997@konkuk.ac.kr; 2 National Agrobiodiversity Center, National Institute of Agricultural Sciences, Rural Development Administration, Jeonju 54874, Korea; awraris@korea.kr

**Keywords:** polyunsaturated fatty acids (PUFAs), erucic acid, petroselinic acid, fat quality indices, hypocholesterolemic fatty acids, atherogenic index (AI)

## Abstract

Spices and herbs are well-known for being rich in healthy bioactive metabolites. In recent years, interest in the fatty acid composition of different foods has greatly increased. Thus, the present study was designed to characterize the fatty acid composition of 34 widely used spices and herbs. Utilizing gas chromatography (GC) flame ionization detection (FID) and GC mass spectrometry (MS), we identified and quantified 18 fatty acids. This showed a significant variation among the studied spices and herbs. In general, oleic and linoleic acid dominate in seed spices, whereas palmitic, stearic, oleic, linoleic, and α-linolenic acids are the major constituents of herbs. Among the studied spices and herbs, the ratio of *n*−6/*n*−3 polyunsaturated fatty acids (PUFAs) was recorded to be in the range of 0.36 (oregano) to 85.99 (cumin), whereas the ratio of PUFAs/saturated fatty acids (SFAs) ranged from 0.17 (nutmeg) to 4.90 (cumin). Cumin, coriander, fennel, and dill seeds represent the healthiest fatty acid profile, based upon fat quality indices such as the ratio of hypocholesterolemic/hypercholesterolemic (h/H) fatty acids, the atherogenic index (AI), and the thrombogenic index (TI). All these seed spices belong to the Apiaceae family of plants, which are an exceptionally rich source of monounsaturated fatty acids (MUFAs) in the form of petroselinic acid (C18:1n12), with a very small amount of SFAs.

## 1. Introduction

Spices and herbs are a vital part of human nutrition around the world, especially in India, China, and southeastern Asian countries [1]. Spices and herbs are food adjuncts, traditionally used as flavoring, seasoning, coloring, and as a food preservative agent [1,2]. Moreover, spices and herbs are an exceptionally rich source of nutritionally important phenolic compounds [3]. These phenolic compounds are primarily responsible for the potent antioxidative, digestive stimulative, hypolipidemic, antibacterial, anti-inflammatory, antiviral, and anticancer properties of spices and herbs [4,5,6].

In general, the terms herbs and spices have more than one meaning. However, the most widely used are those that consider herbs to be derived from the green parts of a plant, such as a stem and leaves used in small amounts to impart flavor, whereas spices are obtained from seeds, buds, fruits, roots, or even the bark of the plants [2].

Fatty acids are the primary nutritional components found in edible seed oils [7]. Seed oils provide essential polyunsaturated fatty acids, linoleic acid (*ω*−6 or *n*−6), and α-linolenic acid (*n*−3) to humans and other higher animals. In the human body, linoleic acid give rise to *n*−6 very long-chain (VLC)-PUFA arachidonic acid, and α-linolenic acid converts to *n*−3 VLC-PUFA eicosapentaenoic acid (EPA), and docosahexaenoic acid (DHA, *n*−3). These *n*−6 and *n*−3 VLC-PUFAs plays key distinct roles in regulating body homeostasis. In general, *n*−6 VLC-PUFAs gives rise to proinflammatory mediators (eicosanoids) whereas *n*−3 VLC-PUFAs give rise to anti-inflammatory mediators. Thus, a higher amount of *n*−3 VLC-PUFAs in the body may protect from chronic diseases, including cancer, inflammatory, or cardiovascular diseases (CVD) [8]. Moreover, a diet with a high proportion of *n*−6 PUFAs (high ratio of *n*−6/*n*−3 PUFAs) cannot be considered beneficial to health, as *n*−3 PUFAs to *n*−3 VLC-PUFAs conversion occurs at a very low rate (e.g., 8% for EPA and less than 1% for DHA), and conversion is largely dependent upon the ratio of ingested *n*−6 (linoleic acid) and *n*−3 (α-linolenic) PUFAs [9]. In human hepatoma cells, this conversion is highest when these *n*−6 and *n*−3 acids are provided at a 1:1 ratio. Thus, the consumption of an appropriate amount of fats with a 1:1 *n*−6/*n*−3 PUFAs ratio, which was probably followed by our ancestors [10], may be considered beneficial.

Similar to the consumption of fats with a balanced ratio of *n*−6/*n*−3 PUFAs, growing evidence suggests that replacing saturated fatty acids (SFAs) with monounsaturated fatty acids (MUFAs) from plant sources may decrease the risk of CVD [11]. And with the health benefits associated with consumption of *n*−3 PUFAs and MUFAs, consumer interest is shifting towards foods with a low proportion of SFAs, a high proportion of MUFAs, and balanced *n*−6/*n*−3 PUFAs. Given this, it is necessary to characterize all the major and minor components of the diet to acquire a better estimate of the fatty acid composition of our food.

Spices and herbs are not a significant source of fatty acids, as they form a small part of the diet. However, a detailed and comparative study of the fatty acid composition of various spices and herbs may be useful to identify those with health-beneficial fatty acids. Considering these facts, this study aims to investigate the fatty acid composition of commercially available major spices and herbs utilizing gas chromatography-flame ionization detection and GC-mass spectrometry analysis. We used fatty acid composition data to study spices and herbs to determine their fat quality indices. We anticipate the results contained herein will contribute significantly to the identification of spices with a healthy fatty acid profile.

## 2. Materials and Methods

### 2.1. Plant Material, Reagents, and Standards

A total of 34 commercially packed spices and herbs (Table 1; 200–500 g each spice and herb from at least three different brands) were obtained from retail outlets in Seoul, Korea. The spice and herb samples of different brands were mixed in equal proportions (200–300 g total) to make a representative sample, ground into a fine powder using a 7010HG laboratory blender (Waring Commercial, Torrington, CT, USA), placed into an air-tight container, and stored at room temperature. The fatty acid standard mix (37 Component FAME Mix, CRM47885) was obtained from Merck Ltd., Seoul, Korea. The organic solvents used for the extraction of lipids were of high-pressure liquid chromatography (HPLC) grade, obtained from Samchun Chemical Co., Ltd., Seoul, Korea.

### 2.2. Extraction of Crude Lipid Compounds

The crude lipids were extracted by using the previous method [12,13] with minor modification. Briefly, 0.6 g dehydrated and powdered spices and herb samples were precisely weighed and transferred to a 50 mL glass tube. In each tube, 150 mg sodium ascorbate and 22 mL (isopropyl alcohol/cyclohexane, 10:12, *v*/*v*) containing 0.075% butylated hydroxytoluene (BHT: *w*/*v*; antioxidant) were added, and the contents were subjected to bath sonication (JAC-2010; 300 w, 60 Hz, for 12 min) for efficient disintegration and complete extraction, followed by 15 h shaking (200 RPM at 22 °C) in a rotary shaker. Contents were centrifuged at 7000× *g* (12 min at 4 °C). The supernatant was collected, and pellets were extracted again with 30 mL cyclohexane. Supernatants from both extractions were pooled (total volume of ~50 mL) and partitioned with an equal volume of 1 M of sodium chloride (NaCl). The upper cyclohexane phase containing crude lipids were collected, filtered over anhydrous sodium sulfate, transferred to a 250-mL round-bottom flask, and vacuum-dried in a rotary evaporator at 30 °C. The crude lipids were recovered into 3 mL methanol/dichloromethane (DCM) (1:3, *v*/*v*) containing 0.1% BHT, transferred to a 5 mL glass vial fitted with a Teflon-lined screw cap, and stored at −20 °C. One milliliter of sample was used to prepare fatty acid methyl esters (FAMEs).

### 2.3. Preparation of Fatty Acid Methyl Esters (FAMEs)

The crude lipids extracted from the spices and herb samples were used to prepare the FAMEs, following the previously optimized method [14] with minor modification. Briefly, 1 mL of a crude lipids sample was transferred into a 5 mL glass vial fitted with a Teflon-lined screw cap. Contents were evaporated to dryness using a rotary evaporator at 30 °C. After evaporation, 3 mL of anhydrous methanolic-HCl (methanol/acetyl chloride, 95:5, *v*/*v*) was added and incubated for 2 h at 55 °C in a heat block. Samples were cooled in ice, and FAMEs were sequentially washed with 1M NaCl and 2% sodium bicarbonate (NaHCO_3_) and recovered in 4 mL hexane. A pinch of anhydrous sodium sulfate (Na_2_SO_4_) was added to the recovered sample (hexane) to absorb the traces of water. One milliliter of sample was filtered through a 0.45 μm PTFE syringe filter and transferred to a 1.5 mL autosampler vial for GC-FID and GC-MS analysis.

### 2.4. GC-FID and GC-MS Analysis of FAMEs

FAMEs were quantitatively analyzed with GC (Agilent 7890B, Agilent Technologies Canada, Inc., Mississauga, ON, Canada) equipped with an autoinjector, an FID, and an SP-2560 capillary column (100 m, 0.20 μm film thickness, 0.25 mm ID; Merck KGaA, Darmstadt, Germany). The injector and the detectors were maintained at 250 °C and 260 °C, respectively. The inlet flow was 2 mL/min with a constant pressure of 54 psi. The FID parameters of hydrogen (H_2_) fuel flow, airflow, and make flow (nitrogen, N_2_) were set to 30, 400, and 25 mL/min, respectively. The column oven temperature was kept at 140 °C for 5 min, then progressively increased to 240 °C for 25 min (linear temperature program 4 °C/min and held at 240 °C for 15 min [15]. The FAMEs were precisely identified by comparing them with the retention time with authentic standards. For a more accurate qualitative analysis, the mass spectra were also recorded using a GC-MS system (QP2010 SE; Shimadzu, Kyoto, Japan), following the optimized GC-FID analysis thermal program. The identity of FAMEs was confirmed by comparing their fragmentation pattern with authentic standards, and also by using the National Institute of Standards and Technology (NIST; U.S. Department of Commerce, Gaithersburg, MD, USA) mass spectrum database (NIST08 and NIST08s).

### 2.5. Calculation of Fat Quality Indices

We used the spice and herbs fatty acid profile to determine several nutritional parameters of lipids, including the ratios of PUFAs/monounsaturated fatty acids (MUFAs), PUFAs/saturated fatty acids (SFAs), the ratio of hypocholesterolemic/hypercholesterolemic (h/H) fatty acids, atherogenic index (AI), and thrombogenic index (TI) [16]. The ratio of h/H fatty acids, AI, and TI was calculated with the following equations [16]:

$$h/H=\frac{cis\;C18:1+\sum \mathrm{MUFAs}+\sum \mathrm{PUFAs}}{C12:0+C14:0+C16:0}$$

$$\mathrm{AI}=\frac{C12:0+\left(4\;\times \;C14:0\right)+C16:0}{\sum \mathrm{MUFAs}+\sum \mathrm{PUFAs}}$$

$$\mathrm{TI}=\frac{C14:0+C16:0+C18:0}{\left(0.5\;\times \;\sum \mathrm{MUFAs}\right)+\left(0.5\;\times \;\sum n-6\;\mathrm{PUFAs}\right)+\left(3\;\times \;\sum n-3\;\mathrm{PUFAs}\right)+\left(\frac{\sum n-3\;\mathrm{PUFAs}}{\sum n-6\;\mathrm{PUFAs}}\right)}$$

### 2.6. Statistical Analysis and Quality Control

We performed a total of six replicate extractions and analyses from each representative sample. The data were analyzed by one-way analysis of variance (ANOVA), and homogenous subsets (mean separation) were determined using Turkey HSD with a significance level of *p* < 0.05, utilizing the IBM statistical 25.0 software.

The method used for GC-FID quantification of FAMEs was validated recently [15].

## 3. Results and Discussion

### 3.1. Fatty Acids Composition

**Figure 1:**
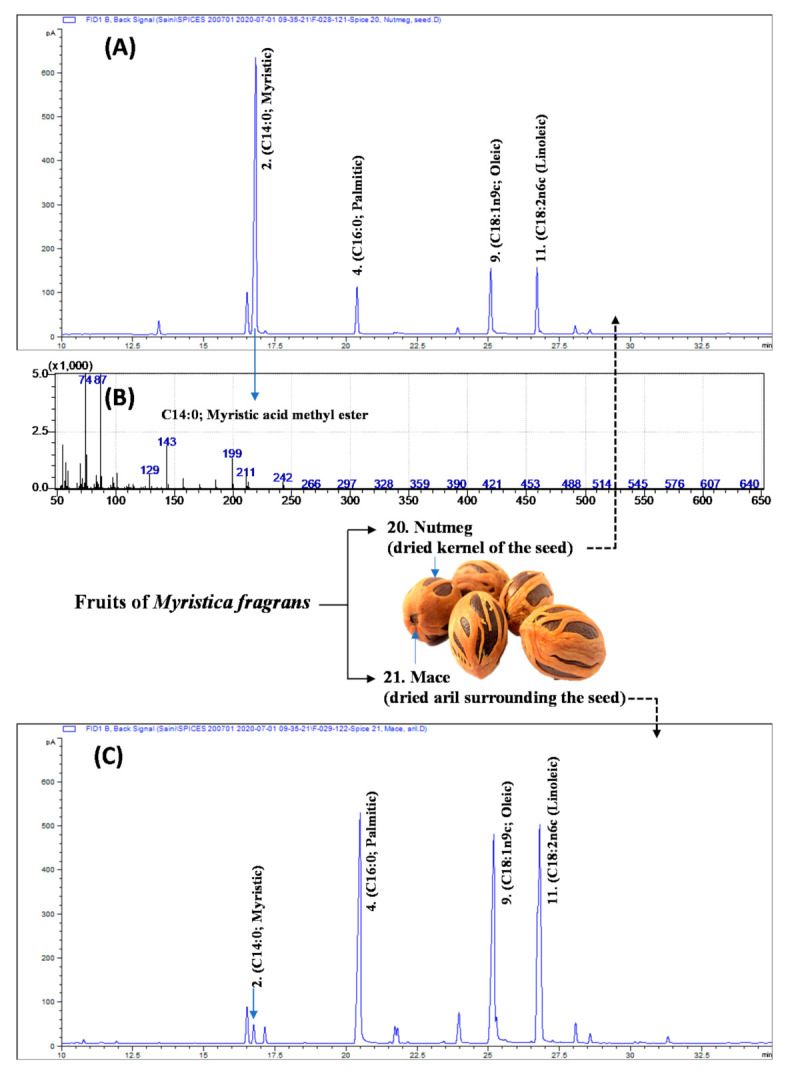
(**A**) The gas chromatography (GC)-flame ionization detection (FID) profiles of fatty acid methyl esters (FAMEs) of nutmeg. (**B**) The GC-mass spectrum of dominating fatty acid (myristic acid) from nutmeg. (**C**) The GC-FID profiles of FAMEs of mace. The numbers, 2, 4, 6, and 8 correspond to peak numbers illustrated in Table 1.

In the present study, 18 fatty acids were identified and quantified, utilizing GC-FID and GC-MS analyses (Table 2). The results, given in Table 2, show that oleic (C18:1n9) and linoleic acid (C18:2n6) are dominated in seed spices, and palmitic (C16:0), stearic, oleic, linoleic, and α-linolenic acid (C18:3n3) are the major constituents of herbs. An exception was myristic (C14:0) acid, which was 60.8% of total fatty acids in *Myristica fragrans* (nutmeg) seeds (Figure 1A,B). Surprisingly, myristic acid was just 1.59% of the total fatty acids in the *M. fragrans* (mace; Figure 1C) seed arils. The highest proportions of oleic acid (41.64–41.85%) were recorded in cardamon pods/capsules (Figure S1) and white pepper seeds (Table 2). The data of the fatty acid composition of cardamom pods and white pepper seeds are scarce. However, 40.6–49.2% of oleic acid has been reportedly extracted from cold-pressed cardamom seeds [17,18], which agrees with data obtained in the present study from whole cardamon pods.

In the present study, a substantial amount of erucic (C22:1n9; 17.3%) and eicosenoic (20:1n9; gondoic acid; 8%) acids were exclusively recorded in white mustard (Sinapis alba; syn Brassica alba) seeds. Similarly, a significant amount of petroselinic acid (C18:1n12c; an isomer of oleic acid) was recorded only in Apiaceae family seeds.

Among the studied 34 spices and herbs, total fatty acids were recorded to be in the range of 2.3 (galangal root) to 130.32 mg/g (mace). The odd chain fatty acid, pentadecanoic (C15:0) acid, was recorded as being a minor constituent (1.18%) in the galangal root. Similarly, heptadecanoic (C17:0) was recorded at only 0.13–0.14% in cayenne pepper, allspice, and mace. In nutmeg (*Myristica fragrans*) seed hexane extract, Anaduaka et al. [19] reported a significant amount of (27%) heptadecanoic (C17:0; margaric) acid. However, in the present study, heptadecanoic acid is not detected in nutmeg seeds.

### 3.2. Black Pepper and White Pepper

Black pepper and white pepper are prepared from the fruits of *Piper nigrum* L., according to the harvesting time and inclusion of the outer skin. Black pepper is the dried immature but fully developed fruit, whereas white pepper consists of the mature fruit lacking the outer skin [20]. The fatty acid composition data of black and white pepper is scarce. In the present study, 28.57%, 14.95%, 26.61%, and 9.32% of palmitic, oleic, linoleic, and α-linolenic acid were recorded being in black pepper. In contrast, 22.55%, 41.64%, 17.19%, and 1.49% of palmitic, oleic, linoleic, and α-linolenic was reported as being in white pepper (Table 2). These observations show that oleic acid increases significantly, whereas the palmitic, linoleic, and α-linolenic acids decrease significantly during the maturation of pepper fruits.

### 3.3. Nutmeg and Mace

Nutmeg and mace spices are obtained from different parts of the same fruit of the nutmeg (*Myristica fragrans*; Myristicaceae) tree. Nutmeg is the dried kernel of the seed, whereas mace is the dried aril surrounding the seed [21]. Myristic acid’s name is derived from *Myristica fragrans*, from which it was first isolated [22]. In the present study, myristic acid was 60.8% of total fatty acids in nutmeg, followed by oleic (C18:1n9c; 13.4%), linoleic (C18:2n6c; 11.9%), and palmitic (C16:0; 8.94%) (Figure 1A). Surprisingly, in mace, linoleic acid was 33.7% of total fatty acids, followed by palmitic (30.6%) and oleic (28.0%). Myristic acid was only 1.59% of the total fatty acids (Figure 1C, Table 2). In the investigations of Al-Khatib et al. [23], myristic acid was recorded as being 79.7% of the total fatty acids in nutmeg. Kozłowska et al. [24] analyzed the fatty acids composition of plant seeds, including anise, coriander, caraway, white mustard, and nutmeg. They reported dominance of oleic (56.5%), palmitic (18.29%), and linoleic (13.6%) acids in nutmeg. These contrasting observations probably arose as these authors reported only above C16 fatty acids. Myristic acid is widely used in the food industry as a flavor ingredient. It is approved as a pharmaceutical excipient by the Food and Drug Administration (FDA) and declared generally recognized as safe (GRAS) by various regulators [25].

### 3.4. Erucic Acid in White Mustard

**Figure 2:**
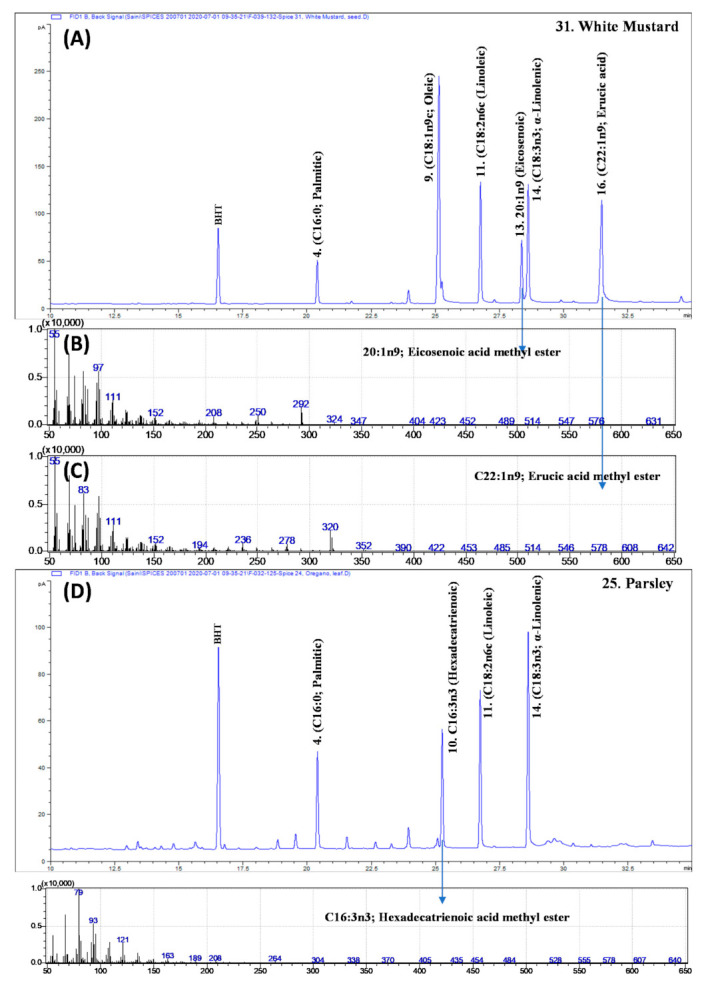
(**A**) The gas chromatography (GC)-flame ionization detection (FID) profiles of fatty acid methyl esters (FAMEs) of white mustard seeds. (**B**,**C**) The GC-mass spectrum of eicosenoic acid and erucic acid from white mustard seeds. (**D**) The GC-FID profiles of FAMEs of parsley leaves. The numbers, 4, 9, 10, 13, 14, and 16 correspond to peak numbers illustrated in Table 1. BHT: Butylated hydroxytoluene (A synthetic antioxidant used during lipid extraction).

Mustard (*Sinapis alba*; syn *Brassica alba*) seeds are well known for the occurrence of a substantial amount of erucic and eicosenoic acid [24]. In the present study, white mustard seeds were found containing 17.3% and 8.0% of erucic and eicosenoic acid, respectively (Figure 2A, Table 2). High intake of erucic acid is considered harmful for cardiac health [26]. The panel on contaminants in the food chain established a tolerable daily intake (TDI) of 7 mg/kg body weight (BW) for erucic acid based on a no-observed adverse effect level (NOAEL) for myocardial lipidosis in rats and pigs [26]. Considering the 43 mg of total fatty acids/g of white mustard seeds, consumption of 100 g of seeds may provide 7.31 mg of erucic acid. The intake of erucic acid from white mustard used as food condiments in daily food preparations is far below the TDI and is safe for consumption.

Petroselinic acid (C18:1n12c; an isomer of oleic acid) is the major component of the lipid constituent of Apiaceae family seeds [27,28]. In a previous study [27] of dill (*Anethum graveolens*) seeds, 87.2% of total fatty acids were composed of petroselinic acid. Similarly, in celery (*Apium graveolens*), coriander seeds (*Coriandrum sativum*), and fennel seeds (*Foeniculum vulgare*), petroselinic acid was recorded as being 56.1%, 72.8%, and 31.32% of total fatty acids. In agreement with the present study, we have also recorded the 50.4%, 49.4%, 62.1%, and 63.3% of petroselinic acid in dill, coriander celery, and fennel seeds, respectively (Table 2). And a similar high amount of petroselinic acid was reported to be in the seeds of other Apiaceae family plants, such as caraway (*Carum carvi*, 34.1%) and cumin (*Cuminum cyminum*; 49.9%). In seeds of different varieties of caraway, Reiter et al. [28] recorded 33.5–42.5% of petroselinic acid, which is in agreement with the present study. Petroselinic acid possesses potent anti-inflammatory and antiaging properties by reducing the metabolites of arachidonic acid [29]. And owing to its anti-aging properties, petroselinic acid is widely used in cosmetics or dermatological compositions [29]. Surprisingly, petroselinic acid was not detected in herbs (leaves) of the Apiaceae family member parsley (*Petroselinum crispum*). In the parsley herb, hexadecatrienoic (C16:3n3) was reported to be 17.7% of the total fatty acids (Figure 2D), whereas no other spices were found to contain this fatty acid. Parsley has been previously classified as a “16:3” plant owing to the presence of a significant amount of hexadecatrienoic acid in photosynthetic tissues, which is part of primitive lipid metabolism [30].

### 3.5. Fat Quality Indices

The present study is based on the fatty acid composition of 34 spices and herbs. We evaluated them for fat quality indices, including the *n*–6/*n*–3 ratio, AI, TI, and h/H fatty acid ratios (Table 3). Among the studied spices and food condiments, the ratio of *n*–6/*n*–3 PUFAs was found to be in the range of 0.36 (oregano) to 85.99 (cumin). In view of health benefits associated with the consumption of *n*−6/*n*−3 PUFAs ratio of 0.5–2.0 (nearest to 1:1), lipids obtained from leaf spices, including tarragon (0.76), bay leaf (1.33), basil (0.55), marjoram (0.75), parsley (0.48), white mustard (0.95), sage (0.86), and thyme (0.52) can be considered to be beneficial. In general, the high occurrence of α-linolenic acids compared to linoleic acid is responsible for the low *n*−6/*n*−3 ratio in leaves (photosynthetic tissue).

In view of the high risk of CVD and other chronic diseases that are associated with the dietary intake of SFAs [11], fats with a PUFAs/SFAs ratio lower than 0.45 are not advised for diet [31]. In the present study, PUFAs/SFAs ratios ranged from 0.17 (nutmeg) to 4.90 (cumin). Low PUFAs/SFAs ratios of 0.17 in nutmeg lipids are the result of the dominance of myristic acid (an SFA; Figure 1A), whereas in the case of cumin, linoleic acid is dominant over SFAs. In addition to the nutmeg, low PUFAs/SFAs ratios (<0.44) were recorded from galangal root (0.29), lemongrass (0.24), rosemary (0.28), and sage (0.38) because of the occurrence of a substantial amount of palmitic acid (Figure S2).

**Figure 3:**
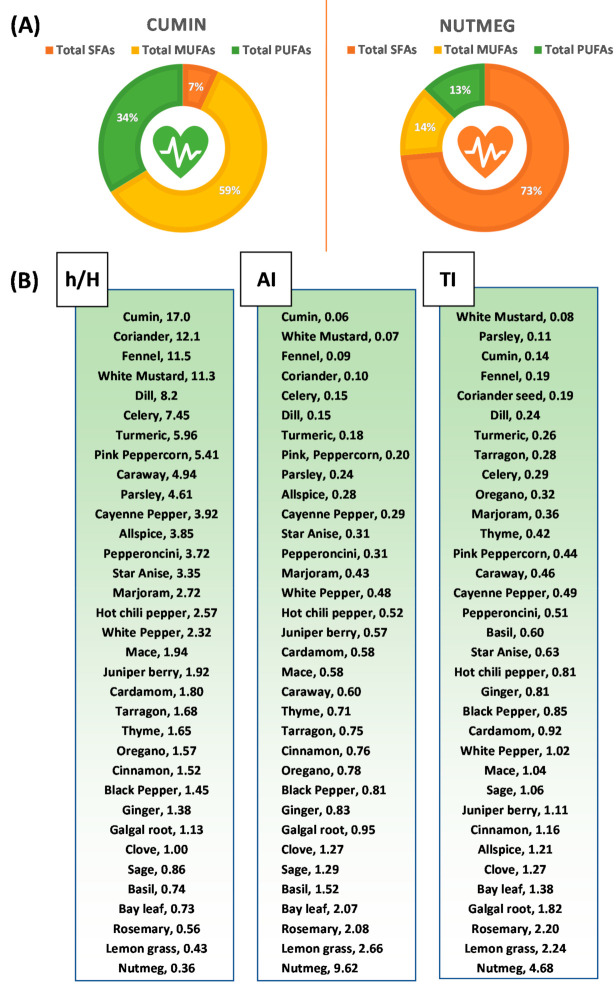
(**A**) Illustrations showing the high content of healthy monounsaturated (MUFAs) and polyunsaturated fatty acids (PUFAs) in cumin, compared to low contents of MUFAs and PUFAs, and high contents of saturated fatty acids (SFAs) in nutmeg. (**B**) Arrangements of studied spices and herbs in ascending/descending order according to the fat quality indices of the ratio of hypocholesterolemic (h)/hypercholesterolemic fatty acids, atherogenic index (AI), and thrombogenic index (TI).

Fats with lower AI and TI and higher ratios of h/H fatty acids are recommended for minimizing the risk of CVD [32]. In the present study, a significant difference was recorded for AI, TI values as well as h/H fatty acids among the studied spices and herbs. The lowest significant values of the AI (0.06) and the highest ratios of h/H fatty acids (17.0) were obtained from cumin seeds (Table 3, Figure 3), because of the presence of a low amount of atherogenic lauric, myristic, and palmitic fatty acids, and high amounts of hypocholesterolemic C18:1 MUFAs and PUFAs. Whereas the lowest significant values of TI (0.08) were recorded in white mustard, due to the low contents SFAs and high content of PUFAs.

Overall, based on a higher ratio of h/H fatty acids and their lower AI and TI values, cumin, coriander, fennel, and dill spices have the healthiest fatty acid profiles (Figure 3). These spices belong to the Apiaceae family. White mustard also represents a higher ratio of h/H fatty acids and lower values of AI and TI. However, it contains a substantial amount of erucic acid.

In Figure 3, cumin, coriander, fennel, and dill spices top the fat quality indices, the ratio of h/H fatty acids, AI, and TI. However, the occurrence of a very low proportion of α-linolenic acid (a *n*−3 PUFA; 0.35–0.85%) and a fairly good amount of linoleic acid (a *n*–6 PUFA; 19.60–33.34%) in these spices, give rise to the high ratio of *n*–6/*n*–3 PUFAs (24.02–85.99), which is substantially higher than the recommended ratio of 1:1. Considering this, the culinary use of these spices can be recommended with *n*–3 PUFA rich components to obtain the overall *n*–6/*n*–3 PUFAs ratio of 1:1.

Previously, we had analyzed the total phenolic contents (TPC) and antioxidant activities of 39 spices and herbs (including the 34 spices and herbs investigated in the present study) and found that cloves possess the highest antioxidant activities, followed by allspice, cinnamon, oregano, and marjoram [33]. The high antioxidant activities of these spices and herbs were probably the results of the richness of phenolic compounds, as the antioxidant activities showed a good correlation (0.835–0.966) with TPC. In contrast, in the present study, cumin, coriander, fennel, and dill spices showed the healthiest fatty acid profile among the 34 spices and herbs. These observations show that the selection of healthy spices and herbs may vary with nutrient requirements. Thus, in the present study, cumin, coriander, fennel, and dill spices are the recommendations based on the fatty acid profile. However, other spices and herbs might be richer in other health-beneficial dietary components.

## 4. Conclusions

Spices belonging to Apiaceae family plants (cumin, coriander, fennel, and dill) are an exceptionally rich source of monounsaturated fatty acids (MUFAs) in the form of petroselinic acid, a good amount of polyunsaturated fatty acids (PUFAs; linoleic acid), and a small amount of saturated fatty acids. And, with high proportions of MUFAs and PUFAs, the Apiaceae family spices top the fat quality indices, particularly in terms of a higher ratio of hypocholesterolemic/hypercholesterolemic fatty acids, and lower values of the atherogenic index and the thrombogenic index (Figure 3).

## Figures and Tables

**Table 1:** List of spices and herbs used in the present investigation (arranged according to botanical name).

Sample No.	Common Name	Botanical Name	Family	Part
1	Galangal root	*Alpinia galanga* (L.) Willd.	Zingiberaceae	Rhizomes
2	Dill	*Anethum graveolens* L.	Apiaceae	Seeds
3	Celery	*Apium graveolens* L.	Apiaceae	Seeds
4	Tarragon	*Artemisia dracunculus* L.	Asteraceae	Leaves
5	Cayenne pepper	*Capsicum annuum* L.	Solanaceae	Pods
6	Pepperoncini	*Capsicum annuum* L. *var. annuum*	Solanaceae	Pods
7	Hot chili pepper	*Capsicum frutescens* L.	Solanaceae	Pods
8	Caraway	*Carum carvi* L.	Apiaceae	Fruits
9	Cinnamon	*Cinnamomum verum* J.Presl	Lauraceae	Bark
10	Coriander seed	*Coriandrum sativum* L.	Apiaceae	Seeds
11	Cumin	*Cuminum cyminum* L.	Apiaceae	Seeds
12	Turmeric	*Curcuma longa* L.	Zingiberaceae	Rhizomes
13	Lemongrass	*Cymbopogon microstachys* (J. D. Hooker) Soenarko	Poaceae	Leaves
14	Cardamom	*Elettaria cardamomum* (L.) Maton	Zingiberaceae	Pods
15	Fennel	*Foeniculum vulgare* Mill.	Apiaceae	Seeds
16	Star anise	*Illicium verum* Hook.f.	Schisandraceae	Fruits
17	Allspice	*Pimenta dioica* (L.) Merr.	Myrtaceae	Fruits
18	Juniper berry	*Juniperus communis* L.	Cupressaceae	Fruits
19	Bay leaf	*Laurus nobilis* L.	Lauraceae	Leaves
20	Nutmeg	*Myristica fragrans* Houtt.	Myrtaceae	Seeds
21	Mace	*Myristica fragrans* Houtt.	Myrtaceae	Aril
22	Basil	*Ocimum basilicum* L.	Lamiaceae	Leaves
23	Marjoram	*Origanum majorana* L.	Lamiaceae	Leaves
24	Oregano	*Origanum vulgare* L.	Lamiaceae	Leaves
25	Parsley	*Petroselinum crispum* (Mill.) Fuss	Apiaceae	Leaves
26	Black pepper	*Piper nigrum* L.	Piperaceae	Fruits (unripe)
27	White pepper	*Piper nigrum* L.	Piperaceae	Seeds
28	Rosemary	*Rosmarinus officinalis* L.	Lamiaceae	Leaves
29	Sage	*Salvia officinalis* L.	Lamiaceae	Leaves
30	Pink peppercorn	*Schinus mole* L.	Anacardiaceae	Fruits
31	White mustard	*Sinapis alba* L.	Brassicaceae	Seeds
32	Clove	*Syzygium aromaticum* (L.) Merr. and L. M. Perry	Myrtaceae	Flower buds
33	Thyme	*Thymus vulgaris* L.	Lamiaceae	Leaves
34	Ginger	*Zingiber officinale* Roscoe	Zingiberaceae	Rhizomes

**Table 2:** Fatty acid composition of spices and herbs.

Peak No	1	2	3	4	5	6	7	8	9	10	11	12	13	14	15	16	17	18
Component (FAME)	C12:0 (Lauric)	C14:0 (Myristic)	C15:0 (Pentadecanoic)	C16:0 (Palmitic)	C16:1 (Palmitoleic)	C17:0 (Heptadecanoic)	C18:0 (Stearic)	C18:1n12c (Petroselinic)	C18:1n9c (Oleic)	C16:3n3 (Hexadecatrienoic)	C18:2n6c (Linoleic)	C20:0 (Arachidic)	20:1n9 (Eicosenoic)	C18:3n3 (α-Linolenic)	C22:0 (Behenic)	C22:1n9 (Erucic)	C24:0 (Lignoceric)	C24:1n9 (Nervonic)
RT	13.44	16.78	18.58	20.42	21.74	22.20	23.96	25.03	25.10	25.20	26.81	27.29	28.36	28.60	30.38	31.48	33.46	34.57
S1	0.62	0.90	1.18 ^a^	37.65	nd	nd	13.06	nd	28.21	nd	13.59	nd	nd	2.45	1.01	nd	1.33	nd
S2	1.20	0.90	nd	8.48	0.34	nd	1.54	50.37	15.26	nd	20.49	0.23	nd	0.85	0.23	nd	0.12	nd
S3	nd	0.44	nd	11.09	0.21	nd	1.91	49.42	7.48	nd	27.55	0.23	nd	1.32	0.21	nd	0.15	nd
S4	6.95	2.90	nd	24.86	nd	nd	3.03	nd	2.23	nd	24.10	2.21	nd	31.84	1.16	nd	0.72	nd
S5	0.27	0.95	nd	18.25	0.64	0.13	2.73	nd	12.31	nd	61.46	0.38	nd	2.53	0.22	nd	0.12	nd
S6	0.27	1.19	nd	18.71	0.86	nd	3.03	nd	12.68	nd	59.65	0.45	nd	2.75	0.27	nd	0.16	nd
S7	1.18	3.22	nd	21.98	0.85	nd	3.87	21.76	17.43	nd	27.99	0.48	nd	0.68	0.36	nd	0.20	nd
S8	nd	10.62	nd	5.55	0.13	nd	2.95	34.09	11.70	nd	33.70	0.50	nd	0.47	0.21	nd	0.07	nd
S9	Nd	1.77	nd	33.97	nd	nd	7.49	nd	23.25	nd	26.99	nd	nd	4.04	1.13	nd	1.36	nd
S10	0.10	0.45	0.14	6.91	0.28	nd	1.36	62.07	7.13	nd	20.85	nd	nd	0.35	0.23	nd	0.12	nd
S11	0.08	0.10	0.16	5.28	0.29	nd	1.08	49.89	9.21	nd	33.34	nd	nd	0.39	0.12	nd	0.07	nd
S12	2.81	0.40	nd	10.51	0.76	nd	2.67	nd	4.48	nd	72.86 ^a^	nd	nd	4.50	0.32	nd	0.67	nd
S13	5.68	2.74	nd	47.82 ^a^	nd	nd	9.46	nd	6.20	nd	12.29	3.89	nd	5.70	3.64 ^a^	nd	2.57	nd
S14	nd	0.75	nd	32.84	1.58 ^a^	nd	3.19	nd	41.81 ^a^	nd	15.07	0.67	nd	3.44	0.26	nd	0.38	nd
S15	0.46	0.17	nd	7.25	0.21	nd	1.24	63.33 ^a^	6.88	nd	19.60	0.16	nd	0.53	0.10	nd	0.07	nd
S16	1.47	0.26	nd	20.26	0.17	nd	3.74	nd	33.75	nd	39.27	nd	nd	0.71	0.29	nd	0.08	nd
S17	nd	0.29	nd	14.27	nd	0.21 ^a^	27.16 ^a^	nd	13.90	nd	39.58	1.39	nd	2.53	0.47	nd	0.20	nd
S18	0.61	0.92	nd	26.01	nd	nd	12.37	nd	22.41	nd	26.98	4.40 ^a^	nd	3.56	1.50	nd	1.24	nd
S19	4.88	8.60	nd	37.43	nd	nd	5.43	nd	20.33	nd	9.55	1.49	nd	7.19	1.51	nd	3.58 ^a^	nd
S20	2.19	60.81 ^a^	nd	8.94	0.39	nd	1.26	nd	13.36	nd	11.94	0.08	nd	0.76	0.14	nd	0.13	nd
S21	0.08	1.59	nd	30.63	1.36	0.14	3.29	nd	28.00	nd	33.72	0.14	nd	0.81	0.13	nd	0.11	nd
S22	17.47 ^a^	1.97	nd	29.85	nd	nd	7.81	nd	7.56	nd	10.18	4.18	nd	18.57	1.63	nd	0.79	nd
S23	1.20	1.33	nd	21.60	nd	nd	5.39	nd	33.65	nd	13.64	2.63	nd	18.23	1.33	nd	0.99	nd
S24	4.91	2.63	nd	26.85	nd	nd	7.89	nd	6.42	nd	12.49	1.83	nd	35.08 ^a^	1.04	nd	0.87	nd
S25	1.15	0.72	nd	15.01	nd	nd	3.38	nd	2.14	17.74 ^a^	24.49	0.24	nd	33.40	0.64	nd	1.09	nd
S26	4.71	1.93	nd	28.57	nd	nd	11.35	nd	14.95	nd	26.61	0.44	nd	9.32	0.81	nd	1.31	nd
S27	2.62	0.95	nd	22.55	nd	nd	11.25	nd	41.64 ^a^	nd	17.59	nd	nd	1.49	0.81	nd	1.11	nd
S28	nd	2.93	nd	47.85 ^a^	nd	nd	11.39	nd	15.62	nd	7.74	3.65	nd	5.31	3.05	nd	2.45	nd
S29	nd	1.59	nd	42.71	nd	nd	10.67	nd	14.51	nd	10.90	3.09	nd	12.62	2.76	nd	1.14	nd
S30	nd	0.35	nd	14.29	0.63	nd	4.59	nd	21.11	nd	56.56	0.43	nd	1.54	0.26	nd	0.24	nd
S31	0.12	0.14	nd	5.53	0.30	nd	1.77	nd	32.97	nd	15.78	0.43	7.96 ^a^	16.59	0.23	17.28 ^a^	0.09	0.82 ^a^
S32	7.95	3.44	nd	27.93	nd	nd	12.60	nd	4.47	nd	28.69	2.29	nd	5.99	2.39	nd	4.26 ^a^	nd
S33	0.91	1.83	nd	29.78	nd	nd	10.11	nd	11.03	nd	14.62	1.52	nd	28.15	1.18	nd	0.87	nd
S34	3.85	1.79	nd	31.47	nd	nd	10.01	nd	7.49	nd	32.47	nd	nd	11.10	0.75	nd	1.07	nd

Values (% of total fatty acids) are the mean of six determinations from each representative sample. RT: retention time. The letter “a” within a column represents the highest significant (*p* < 0.05) values. nd: not detected. Sample numbers (S1–S34) correspond to Table 1.

**Table 3:** The fat quality indices of lipids of spices and herbs.

Sample No	Total FA (mg/g DW)	Total SFAs	Total MUFAs	Total PUFAs	PUFAs: SFAs	PUFAs: MUFAs	*n*−3 PUFA	*n*−6 PUFA	*n*−6/*n*−3	h/H	AI	TI
S1	2.30^b^	55.75	28.21	16.04	0.29	0.57	2.45	13.59	5.55	1.13	0.95	1.82
S2	23.30	12.70	65.97	21.34	1.68	0.32 ^b^	0.85	20.49	24.02	8.22	0.15	0.24
S3	27.60	14.02	57.11	28.88	2.06	0.51	1.32	27.55	20.82	7.45	0.15	0.29
S4	8.30	41.83	2.23^b^	55.94	1.34	25.13	31.84	24.10	0.76 ^b^	1.68	0.75	0.28
S5	120.83	23.06	12.96	63.99	2.78	4.94	2.53	61.46	24.30	3.92	0.29	0.49
S6	48.72	24.07	13.54	62.40	2.59	4.61	2.75	59.65	21.71	3.72	0.31	0.51
S7	42.00	31.29	40.04	28.67	0.92	0.72	0.68	27.99	41.12	2.57	0.52	0.81
S8	103.38	19.90	45.93	34.17	1.72	0.74	0.47 ^b^	33.70	71.19	4.94	0.60	0.46
S9	2.78	45.73	23.25	31.03	0.68	1.33	4.04	26.99	6.68	1.52	0.76	1.16
S10	57.06	9.30	69.49 ^a^	21.21	2.28	0.31 ^b^	0.35 ^b^	20.85	58.96	12.12	0.10	0.19
S11	36.37	6.88 ^b^	59.39	33.73	4.90 ^a^	0.57	0.39 ^b^	33.34	85.99 ^a^	17.01 ^a^	0.06 ^b^	0.14
S12	7.01	17.39	5.24	77.36 ^a^	4.45	14.75	4.50	72.86 ^a^	16.20	5.96	0.18	0.26
S13	2.75	75.80 ^a^	6.20	17.99	0.24	2.90	5.70	12.29	2.16	0.43 ^b^	2.66	2.24
S14	8.87	38.10	43.39	18.50	0.49	0.43 ^b^	3.44	15.07	4.38	1.80	0.58	0.92
S15	53.18	9.45	70.42 ^a^	20.13	2.13	0.29 ^b^	0.53 ^b^	19.60	37.30	11.47	0.09	0.19
S16	29.96	26.11	33.92	39.98	1.53	1.18	0.71	39.27	55.10	3.35	0.31	0.63
S17	22.49	43.98	13.90	42.11	0.96	3.03	2.53	39.58	15.62	3.85	0.28	1.21
S18	5.98	47.05	22.41	30.54	0.65	1.36	3.56	26.98	7.58	1.92	0.57	1.11
S19	5.39	62.92	20.33	16.74	0.27	0.82	7.19	9.55	1.33	0.73	2.07	1.38
S20	61.04	73.56	13.74	12.70 ^b^	0.17 ^b^	0.92	0.76	11.94	15.73	0.36 ^b^	9.62 ^a^	4.68 ^a^
S21	130.32 ^a^	36.12	29.36	34.53	0.96	1.18	0.81	33.72	41.69	1.94	0.58	1.04
S22	6.23	63.69	7.56	28.75	0.45	3.80	18.57	10.18	0.55 ^b^	0.74	1.52	0.60
S23	8.09	34.48	33.65	31.87	0.92	0.95	18.23	13.64	0.75 ^b^	2.72	0.43	0.36
S24	8.84	46.01	6.42	47.57	1.03	7.41	35.08	12.49	0.36 ^b^	1.57	0.24	0.32
S25	14.05	22.23	2.14^b^	75.63	3.40	35.37 ^a^	51.14 ^a^	24.49	0.48 ^b^	4.61	0.78	0.11
S26	5.38	49.12	14.95	35.93	0.73	2.40	9.32	26.61	2.86	1.45	0.81	0.85
S27	3.75	39.28	41.64	19.08	0.49	0.46	1.49	17.59	11.80	2.32	0.48	1.02
S28	4.38	71.32	15.62	13.06 ^b^	0.18 ^b^	0.84	5.31	7.74 ^b^	1.46	0.56	2.08	2.20
S29	6.74	61.96	14.51	23.53	0.38	1.62	12.62	10.90	0.86 ^b^	0.86	1.29	1.06
S30	32.31	20.16	21.74	58.10	2.88	2.67	1.54	56.56	36.78	5.41	0.20	0.44
S31	43.00	8.30	59.33	32.37	3.90	0.55	16.59	15.78	0.95 ^b^	11.29	0.07 ^b^	0.08 ^b^
S32	7.13	60.86	4.47	34.68	0.57	7.76	5.99	28.69	4.79	1.00	1.27	1.27
S33	5.95	46.20	11.03	42.77	0.93	3.88	28.15	14.62	0.52 ^b^	1.65	0.71	0.42
S34	5.26	48.94	7.49	43.57	0.89	5.81	11.10	32.47	2.93	1.38	0.83	0.81

Values are the mean of six determinations. PUFAs: total polyunsaturated fatty acids; MUFAs: total monounsaturated fatty acids; SFAs: total saturated fatty acids; AI: atherogenic index; and TI: thrombogenic index; h/H: ratios of hypocholesterolemic (h)/hypercholesterolemic (H) fatty acids. The letters a and b within a column represent the highest and lowest significant (*p* < 0.05) values, respectively. Sample numbers (S1–S34) correspond to the Table 1.

## Supplementary Materials

The following are available online at https://www.mdpi.com/article/10.3390/foods10040854/s1, Figure S1: (A) The gas chromatography (GC)-flame ionization detection (FID) profiles of fatty acid methyl esters (FAMEs) of cardamom. (B) The GC-mass spectrum of dominating fatty acid (Palmitic acid); Figure S2. (A–C) The gas chromatography (GC)-flame ionization detection (FID) profiles of fatty acid methyl esters (FAMEs) of lemongrass, rosemary, and Sage. The GC-mass spectrum of dominating fatty acid (Palmitic acid). The numbers, 4, 7, 9, 11, and 14 correspond to peak numbers illustrated in Table 1. BHT: Butylated hydroxytoluene (A synthetic antioxidant used during lipid extraction).
