# Supplementary material for: Spices in the Apiaceae Family Represent the Healthiest Fatty Acid Profile: A Systematic Comparison of 34 Widely Used Spices and Herbs
Source: Foods. 2021 Apr 14;10(4):854. doi: 10.3390/foods10040854 (PMC8071036; doi:10.3390/foods10040854)

Supplementary material

Figure S1. (A) The gas chromatography (GC)-flame ionization detection (FID) profiles of fatty acid methyl esters (FAMEs) of cardamom. (B) The GC-mass spectrum of dominating fatty acid (Palmitic acid). The numbers, 4, 7, 9, 11, and 14 correspond to peak numbers illustrated in Tables 1. BHT: Butylated hydroxytoluene (A synthetic antioxidant used during lipid extraction).

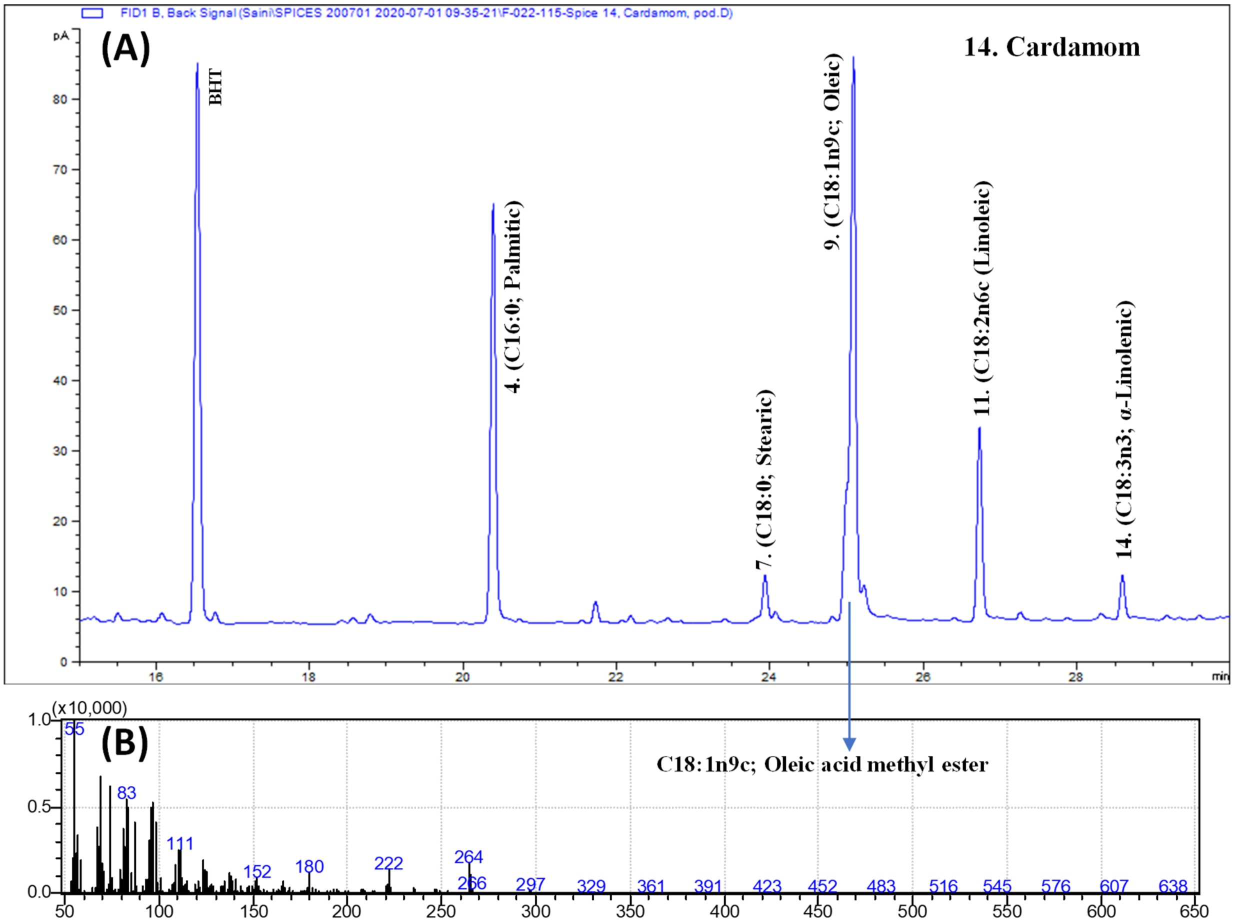

Figure S2. (A-C) The gas chromatography (GC)-flame ionization detection (FID) profiles of fatty acid methyl esters (FAMEs) of lemongrass, rosemary, and Sage. The GC-mass spectrum of dominating fatty acid (Palmitic acid). The numbers, 4, 7, 9, 11, and 14 correspond to peak numbers illustrated in Tables 1. BHT: Butylated hydroxytoluene (A synthetic antioxidant used during lipid extraction).

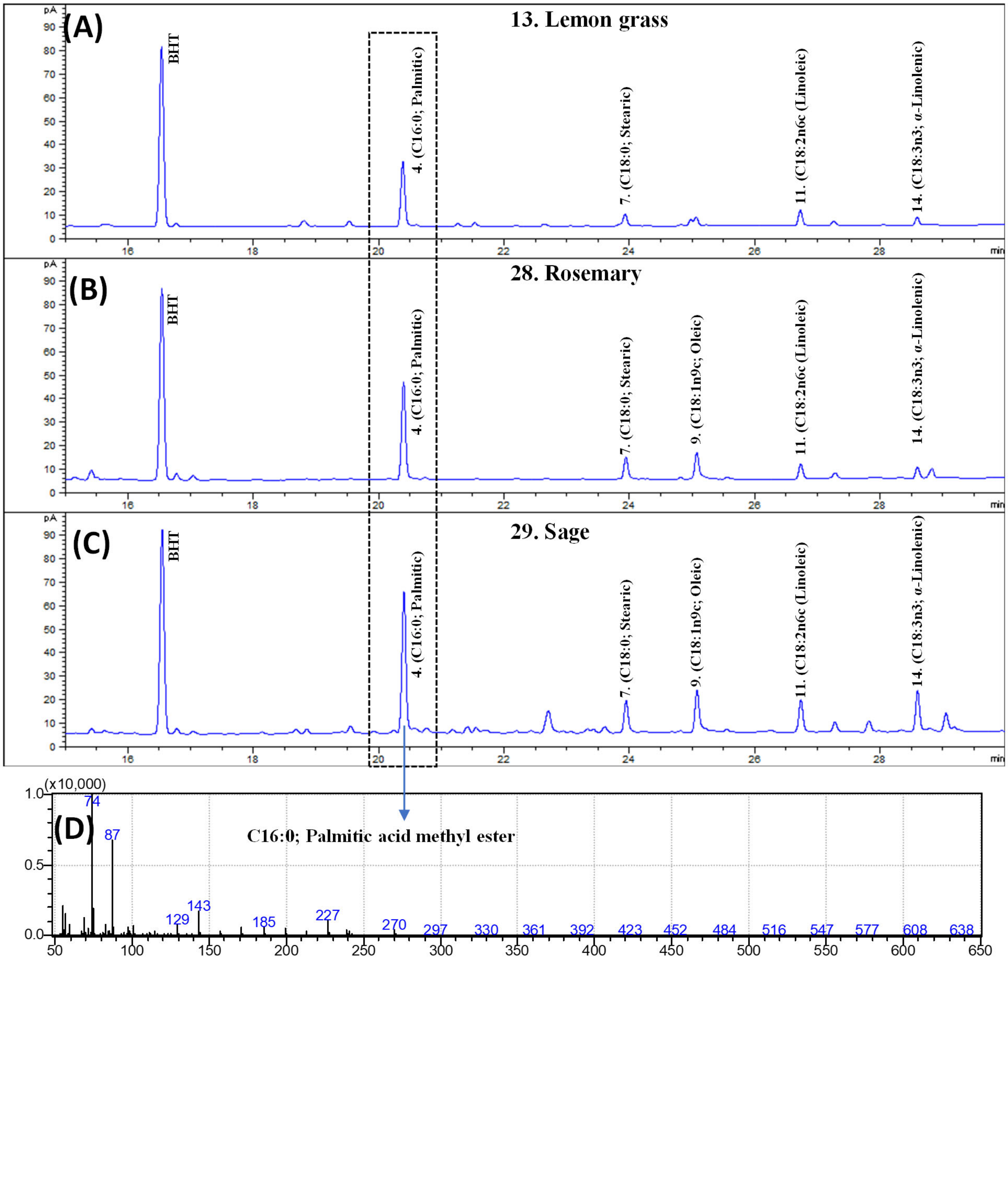

Supplement: Supplementary file 1 [file foods-10-00854-s001.pdf]
